# Síndrome cutáneo-hemorrágico por contacto con orugas de *Lonomia* spp.: primer reporte de la Amazonía ecuatoriana

**DOI:** 10.7705/biomedica.7532

**Published:** 2025-08-11

**Authors:** Manuel Calvopina, Elías Guamán-Charco, Jasmín Vélez, Belén Vélez, Camila González

**Affiliations:** 1 One Health Research Group, Facultad de Medicina, Universidad de las Américas, Quito, Ecuador Universidad de las Américas Universidad de las Américas Quito Ecuador; 2 Hospital “Marco Vinicio Iza”, Ministerio de Salud Pública del Ecuador, Nueva Loja, Sucumbíos, Ecuador Ministerio de Salud Pública del Ecuador Ministerio de Salud Pública del Ecuador Nueva Loja Sucumbíos Ecuador; 3 Departamento de Ciencias Biológicas, Centro de Investigaciones en Microbiología y Parasitología Tropical - CIMPAT, Universidad de los Andes, Bogotá, D. C., Colombia Universidad de los Andes Universidad de los Andes Bogotá, D. C. Colombia

**Keywords:** larva, dermatología, ecosistema amazónico, Ecuador, Larvae, dermatology, Amazonian ecosystem, Ecuador

## Abstract

Se informa el primer caso documentado del síndrome cutáneo hemorrágico -inducido por contacto con orugas venenosas- en una mujer de 29 años, residente en el norte de la Amazonía ecuatoriana. Los casos reportados de este síndrome son raros y se caracterizan por lesiones dérmicas, hemorragias sistémicas y alteraciones de la coagulación.

La mujer tuvo contacto en el muslo derecho con orugas que reposaban sobre el tronco de un árbol. Esto resultó en irritación y dolor locales, seguidos de cefalea, mareos y vómito. A las 48 horas, presentó equimosis en el sitio de contacto, el cuello, el tórax, el abdomen y las extremidades. La paciente fue hospitalizada. Presentó hemorragia vaginal abundante y prolongación de los tiempos de coagulación, aunque con un número normal de plaquetas. Fue tratada con paracetamol y ácido tranexámico, con lo cual se controló el sangrado y se normalizaron los parámetros de coagulación. Se le dio de alta en buenas condiciones y, a los seis meses de seguimiento, permanecía asintomática.

Aunque en la Amazonía ecuatoriana se registran orugas del género *Lonomia*, este es el primer caso diagnosticado. Se destaca la falta de disponibilidad de suero antilonómico en la región, lo que subraya la necesidad de implementar estrategias para garantizar el acceso al suero, y promover la concienciación médica y comunitaria del síndrome cutáneo hemorrágico por contacto con *Lonomia* spp. en áreas endémicas.

## Abstract (en)

This is the first report of hemorrhagic cutaneous syndrome induced by contact with venomous caterpillars in a 29-year-old woman living in the northern Ecuadorian Amazon. Reported cases of hemorrhagic cutaneous syndrome are rare and are characterized by dermal lesions, systemic bleeding, and coagulation abnormalities.

The woman had contact on her right thigh with caterpillars resting on the trunk of a tree which resulted in local irritation and pain, headache, dizziness, and vomiting. After 48 hours, ecchymosis occurred at the contact site, the neck, thorax, abdomen, and extremities. She was hospitalized and presented abundant vaginal bleeding and extended coagulation times with a normal platelet count. She was treated with paracetamol and tranexamic acid, achieving resolution of the bleeding and normalization of coagulation parameters. The patient was discharged in good condition; at the six-month follow-up, she remained asymptomatic.

Although *Lonomia* spp. caterpillars are recorded in the Ecuadorian Amazon, this is the first reported case. This report highlights the unavailability of antilonomic serum in the region, underlining the need for implementing strategies to guarantee serum access and promoting medical and community awareness of hemorrhagic cutaneous syndrome by contact with *Lonomia* spp. in endemic areas.

El síndrome cutáneo hemorrágico en las regiones neotropicales de Sudamérica es causado por el contacto accidental con orugas venenosas. Se caracteriza por reacciones dermatológicas locales y hemorragias internas que en casos graves pueden llevar a la muerte ^1^^-^^3^. Seis países han reportado estos accidentes: Venezuela, Brasil, Colombia, Argentina, Guyana Francesa y Perú ^3^. En el Ecuador, hasta ahora, no se había registrado ningún caso.

Históricamente se han descrito dos especies de orugas - *Lonomia achelous* y *L*. *obliqua*, pertenecientes a las polillas de la familia Saturniidae- como las principales causantes del síndrome cutáneo hemorrágico. Sin embargo, *L. casanarensis*, *L. orientoandensis*, *L. orientocordillera*, *L. parobliqua* y *L. descimoni*, también han sido identificadas como responsables de envenenamiento en humanos ^4^. *Lonomia orientoandensis* y *L*. *casanarensis* han sido implicadas como causantes de síndromes hemorrágicos en modelos experimentales con ratas ^5^.

Sesenta especies de polillas de la familia Saturniidae, género *Lonomia*, están presentes desde México hasta Argentina. En Ecuador, se han encontrado 14 especies en 169 localidades examinadas; en el norte de la Amazonía del país, específicamente en las provincias de Sucumbíos y Orellana, se ha reportado la presencia de L. madrediosiana ^4^. *Lonomia achelous* está presente en la región amazónica en el norte de Sudamérica, incluido Ecuador ^6^, mientras que *L*. *obliqua* se encuentra en el área no amazónica de Brasil, Paraguay, Argentina y Uruguay ^4^. En Ecuador, las orugas son conocidas por los pobladores como “pachones”.

El diagnóstico del síndrome cutáneo hemorrágico suele basarse en los hallazgos clínicos, el antecedente de contacto con orugas y la residencia en zonas tropicales endémicas para polillas. Inmediatamente después del contacto con orugas *Lonomia* spp., se produce dermatitis urticante. En las horas siguientes, aparecen síntomas inespecíficos como cefalea, malestar general, náuseas, vómitos, ansiedad y mialgias; con menor frecuencia, se ha reportado dolor abdominal, hipotermia e hipotensión. Después de ocho a 72 horas, aparecen hemorragias dérmicas como equimosis en el sitio de contacto y en otros lugares de la piel; también, pueden ocurrir hemorragias sistémicas, hematomas espontáneos o causados por traumatismos, gingivorragia, epistaxis, hematemesis, enterorragia, hipermenorragia, hematuria macroscópica, sangrado de heridas recientes, hemorragias intraarticulares y, en casos graves, hemorragias de órganos vitales, como la intraparenquimatosa cerebral que es una causa de muerte ^7^.

Según su gravedad, el síndrome cutáneo hemorrágico se clasifica como sigue ^7^:

- Leve: presencia de síntomas locales dérmicos, sin alteraciones de la coagulación ni hemorragias durante las 48 horas después del accidente.
- Moderado: síntomas locales, alteraciones de la coagulación (en ocasiones, pueden ser la única manifestación clínica), lesiones hemorrágicas en la piel o las mucosas (equimosis, hematomas o gingivorragia), o hematuria, pero sin cambios hemodinámicos (hipotensión, taquicardia o choque).
- Grave: trastornos de la coagulación y hemorragias viscerales (hematemesis, hipermenorragia, hemorragia pulmonar o intracraneal), cambios hemodinámicos y, en ocasiones, falla orgánica o sistémica múltiple ^7^.

No existen exámenes de laboratorio específicos para diagnosticar el síndrome cutáneo hemorrágico. Sin embargo, ciertos hallazgos pueden sugerir su presencia, como la prolongación de los tiempos de coagulación de la protrombina, la trombina y la tromboplastina parcial activada; el aumento de los productos de degradación del fibrinógeno y la fibrina; y la disminución del tiempo de coagulación del fibrinógeno plasmático. En muchos casos, el número de plaquetas puede permanecer dentro de los rangos normales ^7^.

El tratamiento específico para el síndrome cutáneo hemorrágico es el suero antilonómico producido con antígenos de *L*. *obliqua* y desarrollado por el Instituto Butantan de Brasil ^8^. El suero antilonómico se ha utilizado con éxito para tratar el envenenamiento con diversas especies de *Lonomia* e, incluso, en casos en los que no se logró identificar la especie de oruga^5^^,^^9^^-^^11^.

La dosis del antídoto varía según la gravedad del envenenamiento, como se muestra en el esquema de orientación terapéutica en casos de accidentes por *Lonomia* de la Fundación Nacional de Salud (FUNASA) de Brasil ^7^. En los casos leves, el tratamiento es sintomático, similar al de una dermatitis de contacto, mientras que, para los envenenamientos moderados y graves, se recomienda la administración de 5 y 10 viales del suero, respectivamente. No obstante, el suero antilonómico no está disponible en todas las regiones ni países endémicos como Ecuador. En Colombia, el Instituto Nacional de Salud ha producido un suero polivalente contra *L*. *casanarensis* y *L*. *orientoandensis*, pero aún no se reportan estudios clínicos asociados con el uso de este antiveneno.

Agentes antifibrinolíticos, como el ácido aminocaproico y la aprotinina, se han administrado experimentalmente a ratas envenenadas por *L*. *obliqua*. Sin embargo, no se demostró eficacia terapéutica, por lo que los autores concluyen que debe evitarse su uso como tratamiento ^5^. No obstante, en Venezuela, el ácido aminocaproico está indicado para tratar pacientes afectados por *L*. *achelous*^12^. La administración de sangre total o plasma fresco esta contraindicada en casos de envenenamiento por orugas de *Lonomia* spp., ya que podrían acentuar la coagulación intravascular y disminuir seriamente el número de plaquetas, agravándose así el cuadro clínico. En los casos con anemia, se recomienda administrar concentrados de glóbulos rojos ^7^.

En este estudio, se informa un envenenamiento por contacto con orugas de *Lonomia* spp. en la Amazonía ecuatoriana, y se amplía el conocimiento de su distribución geográfica y presentación clínica en Suramérica.

## Presentación del caso

**Figura 1:**
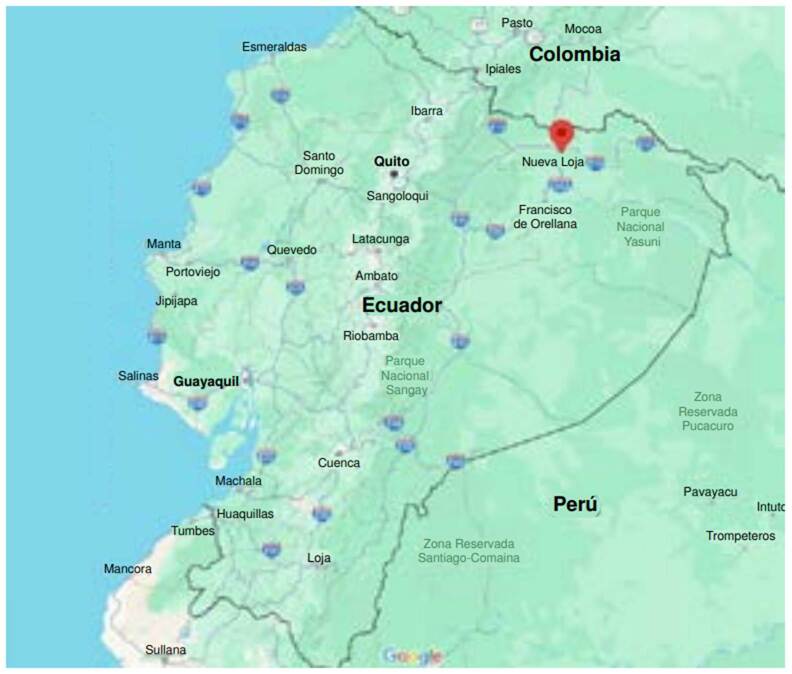
Mapa del Ecuador. El icono rojo indica el lugar donde ocurrió el accidente (0.02609520° N, -76.84259° W), ubicado a 16 km vía El Coca o a 15 minutos en carro de Nueva Loja, capital de la provincia de Sucumbíos, a 33 km de la frontera con Colombia. https://Www.google.eom/maps/@-1.7364579,-78.4711501,7.2z?entry=ttu

Se trata de una mujer de 29 años, residente en la cooperativa “Trabajadores” ubicada al norte de la Amazonía ecuatoriana (figura 1). Acudió a urgencias por presentar un cuadro clínico de 8 horas de evolución, caracterizado por dolor urente y eritema en la parte postero-interna del muslo derecho, tras haber tenido contacto con orugas agrupadas en el tronco de un árbol de guamo, cuyo fruto recibe diversos nombres, como guamas o guabas, en Ecuador, también, jinicuile o mandraque. Luego de 3 a 4 horas del contacto, presentó malestar general, mareo, náuseas y vómito en tres ocasiones y, además, cefalea moderada. El diagnóstico inicial en urgencias fue “picadura de insecto” (CIE-10 W578) y se prescribió paracetamol. Fue dada de alta con la recomendación de capturar las larvas para su identificación, ya que no parecía tratarse de un caso grave. Sin embargo, 56 horas después del accidente, regresó a urgencias por presentar “moretones” (equimosis) en el sitio de contacto con las orugas y en otras regiones del cuerpo, cefalea persistente y mareo. Refirió no haber sufrido traumatismos y negó antecedentes médicos de importancia.

La paciente fue hospitalizada con el diagnóstico de “envenenamiento por orugas”. Se le practicaron exámenes de laboratorio, se mantuvo bajo observación y se dio manejo sintomático. La mujer no manifestó antecedentes médicos de importancia. Ingresó con presión arterial de 126/63 mm Hg, frecuencia cardiaca de 85 latidos por minuto, frecuencia respiratoria de 18 respiraciones por minuto, saturación de oxígeno del 98 % y temperatura de 36,3 ^o^C. Se encontraba orientada en tiempo y espacio (escala de coma Glasgow: 15/15).

**Figura 2:**
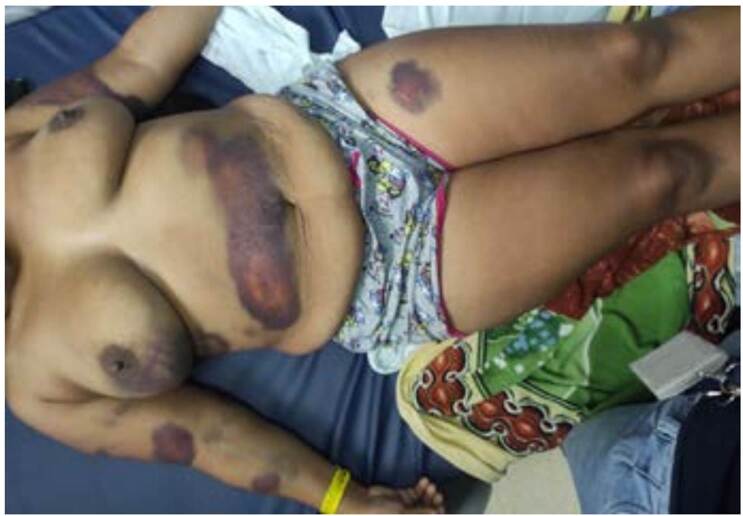
Lesiones cutáneas 56 horas después del contacto con las orugas. Se observan lesiones rojizas, azul oscuras y violáceas en tórax, abdomen, brazos, antebrazos, muslos y piernas. Las lesiones son de tamaño variable: desde pequeñas hasta grandes como las observadas en el brazo derecho y el abdomen. Las lesiones no tienen forma definida, son difusas, de contornos irregulares, hipersensibles y ligeramente dolorosas, elevadas al tacto y sin signos de inflamación ni de infección.

Se observaron lesiones equimóticas en la piel de cuello, tórax, abdomen, región mamaria derecha, glúteo derecho y miembros superiores e inferiores (figura 2). En el examen físico, no se encontraron signos de sangrado en mucosas u otras alteraciones sistémicas.

Al momento del ingreso y durante la consulta de seguimiento, se practicaron exámenes de laboratorio, cuyos resultados y valores de referencia por parámetro se presentan en el cuadro 1. Los tiempos de coagulación (protrombina, tromboplastina parcial e índice internacional normalizado) fueron más prolongados, por lo que se administró fitomenadiona (vitamina K_1_) por vía intravenosa. No se practicaron otras pruebas de coagulación, más específicas, por no disponer de los reactivos en el hospital.

**Cuadro 1:** Resultados de los exámenes de laboratorio de la paciente

Exámenes	Ingreso (24/08/2023)	Día 1	Día 2	Día 3	Control 11/09/2023	Valores de referencia
Tiempo de protrombina (s)	28,1	28,8	28,4	22,7	10,3	10-14
INR	2,89	2,96	2,72	2,20	0,98	0,7-1,1
Tiempo parcial de tromboplastina (s)	78,2	55,7	55,0	38,2	25,1	23,4-38,2
Leucocitos (células/L)	10,15	--	10,8	8,78	7,93	5,0-11,0x10^9^
Basófilos (%)	0,3	--	0,5	0,3	0,4	0-1
Eosinófilos (%)	6,3	--	7,7	9,7	15,8	1-6
Neutrófilos (%)	63,2	--	60,1	54,6	45,4	46-62
Linfocitos (%)	25,3	--	26,1	30,3	32,2	28-44
Monocitos (%)	4,9	--	5,6	5,1	6,2	2-8
Eritrocitos (células/L)	4,51	--	3,17	3,22	4,63	4,3-5,7 x 10^9^
Hemoglobina (g/dl)	12,6	--	9,9	9,8	13,6	13,2-1,8
Hematocrito (%)	40,4	--	31,7	29,4	42,1	40,0-54,0
VCM	89,7	--	89,5	89,5	91,4	72-96
HCM (pg)	28	--	27,9	28,6	30,2	27-32
CHCM (g/dl)	31,2	--	31,2	32	33	31-36
Plaquetas células /μΙ	303	--	273	276	450	150-450

INR: *International Normalized Ratio*; CVM: volumen corpuscular medio; HCM: hemoglobina corpuscular media; CHCM: concentración de hemoglobina corpuscular media

Al siguiente día, la paciente presentó sangrado vaginal anormal y abundante, y disminución de los valores hematimétricos, motivo por el cual se indicó ácido tranexámico por vía oral (1 g cada 8 horas por 2 días). En el examen físico, persistían las equimosis, pero no aumentaron en número o tamaño.

Las pruebas de coagulación continuaron alteradas en los siguientes dos días de hospitalización. Sin embargo, a partir del tercer día empezaron a normalizarse, así como el número total de glóbulos rojos y los valores de hemoglobina y hematocrito (cuadro 1). La paciente no presentó alteraciones hemodinámicas y el sangrado vaginal desapareció al tercer día. Se prescribió omeprazol por vía oral e intravenosa (40 mg una vez al día) para prevenir un posible sangrado gastrointestinal. Al cuarto día de hospitalización, la paciente recibió el alta médica con indicación de control en 15 días.

En la consulta de seguimiento, no refirió cefalea ni náuseas; algunas lesiones equimóticas habían desaparecido y otras presentaban un color verde-amarillento. Los resultados de los exámenes de biometría hemática y las pruebas de coagulación se encontraron dentro de los rangos normales, aunque el porcentaje de eosinófilos resultó elevado (cuadro 1). Seis meses después, en otro control de seguimiento, la paciente se encontraba asintomática.

Los familiares capturaron varias orugas, pero como fueron recogidas dos días después del accidente, se consideró que probablemente no fueron las causantes del envenenamiento y, por lo tanto, no fueron identificadas.

### Consideraciones éticas

La paciente firmó el consentimiento informado para la publicación de su caso con fines académicos.

## Discusión

El primer caso del síndrome cutáneo hemorrágico causado por orugas venenosas fue reportado en 1967 en Venezuela y, posteriormente, se reportaron otros en cinco países sudamericanos, incluyendo un caso reciente en la Amazonía venezolana ^3^^,^^4^. Este es el primer reporte en Ecuador. Los autores de este estudio consideran que hay un subregistro del síndrome cutáneo hemorrágico por desconocimiento del personal de salud, y porque en los casos leves y moderados, a menudo no se busca la atención médica. Lo anterior se debe a las dificultades que enfrentan los pobladores en la Amazonía para acceder a los centros de salud.

La finalidad de reportar este caso de síndrome cutáneo hemorrágico por contacto con orugas venenosas es alertar a la comunidad científica y médica sobre su presencia en la región, e incentivar su inclusión en el diagnóstico diferencial frente a equimosis y sangrados anormales.

En el presente caso no se identificó la especie de oruga responsable; sin embargo, los hallazgos clínicos -como las hemorragias dérmicas tipo equimosis, la hemorragia vaginal anormal, el antecedente de contacto accidental con orugas en el tronco de un árbol y la prolongación de los tiempos de coagulación- son indicativos del síndrome cutáneo hemorrágico. Además, la presencia de la especie venenosa *L*. *achelous* ha sido documentada en la Amazonía ecuatoriana, incluyendo la provincia de Sucumbíos, donde reside la paciente.

Otras orugas del género *Lonomia* identificadas en el área, son *L*. *descimoni*, *L*. *orientoandensis*, *L*. *orientocordillera*, *L*. *canescens* y *L*. *madrediosiana*. Las primeras tres especies se han asociado con el síndrome cutáneo hemorrágico tras contacto con orugas, en los países vecinos de Colombia y Perú ^4^. *Lonomia orientoandensis* y *L*. *casanarensis* también están implicadas en el desarrollo de síndromes hemorrágicos en modelos experimentales con ratas ^5^, y ambas especies están presentes en la región amazónica.

Este reporte busca incentivar futuras investigaciones sobre las especies de orugas y polillas de la familia Saturniidae -y otras relacionadas- presentes en el área de residencia donde ocurrió el presente caso, empleando preferiblemente métodos de identificación molecular ^4^.

El género *Lonomia* es endémico de la región neotropical y cuenta con alrededor de 60 especies distribuidas desde México hasta el norte de Argentina. De la mayoría de estas no se conoce su patogenicidad. Históricamente, solo se han identificado *L*. *obliqua* y *L*. *achelous* como causantes del síndrome cutáneo hemorrágico y, en algunos casos, de muertes. No obstante, otras siete especies se han relacionado con este síndrome, ya que se encuentran en lugares donde se han reportado casos de envenenamiento ^4^. No se conocen casos humanos en México ni en países de Centroamérica, sin embargo, puede haber falta de registro de estos accidentes.

El cuadro clínico que presentó la paciente fue similar a los descritos en la literatura revisada sobre el síndrome cutáneo hemorrágico. Según la clasificación del FUNASA ^7^, la gravedad del presente caso fue moderada y se destacan como hallazgos principales: equimosis en varios sitios de la piel sin sangrado interno aparente (excepto el transvaginal) y sin compromiso hemodinámico; el hematocrito estaba disminuido por la hemorragia y la hemoglobina se encontraba en niveles tolerables, por lo que no se requirió de la trasfusión de paquetes globulares; las plaquetas se mantuvieron dentro de los límites normales como se menciona en otros casos ^7^. La paciente se recuperó completamente sin la necesidad de administrar el suero antilonómico. Sí se administró ácido tranexámico como fármaco antifibrinolítico indicado en casos de sangrado vaginal abundante ^13^.

En Ecuador no está disponible el suero antilonómico, por lo cual se recomienda a las autoridades del Ministerio de Salud Pública solicitarlo y proveerlo a los centros de salud y los hospitales localizados en las áreas endémicas. Este se puede conseguir con el Instituto Butantan (https://butantan.gov.br/) o la 9^a^ Regional de Saúde - Foz do Iguaçu (Rua Santos Dumont, 460, Centro), (+55) (45) 3 545 7100. También, se recomienda la divulgación de este reporte al personal de salud y a la población general para dar a conocer un poco más sobre las manifestaciones clínicas del síndrome cutáneo hemorrágico y las orugas que lo ocasionan en el país. Este estudio puede ser un insumo útil para la generación de estrategias de prevención y control mediante campañas de comunicación masiva.

Se sugiere investigar la eficacia del suero antilonómico desarrollado en Brasil contra el veneno de otras especies de orugas, como aquellas encontradas en la Amazonía ecuatoriana, y revisar la taxonomía de las especies de orugas y polillas implicadas. El envenenamiento por orugas debería considerarse una enfermedad tropical desatendida, así como la mordedura de serpientes, declarada como tal por la Organización Mundial de la Salud (OMS) en el 2017 ^14^.

Es importante mencionar que el sitio del accidente ocurrió en una comunidad ganadera y con plantaciones frutales, a 16 km de Nueva Loja, la ciudad más poblada de la Amazonía ecuatoriana, con 55.627 habitantes, según el censo del 2022. Las especies de orugas venenosas del género *Lonomia* residen en áreas boscosas tropicales, pero su presencia en esta comunidad suburbana sugiere que están siendo atraídas a centros poblados. Este fenómeno podría estar relacionado con la deforestación, el cambio climático y la ocupación humana de los ecosistemas naturales de las orugas. La deforestación causada por el hombre, la introducción de asentamientos iluminados y la siembra de árboles frutales pueden atraer a las polillas en busca de plantas para ovipositar. Esta situación puede crear un problema de salud pública en las áreas urbanas, considerado incluso como una entidad emergente en países como Brasil y Argentina ^9^. Aunque Ecuador tiene protocolos para el manejo del envenenamiento por serpientes y escorpiones ^15^, aún no dispone de guías para el causado por orugas.

Por otro lado, en estas regiones, se debería investigar el impacto de estos incidentes en la ganadería y los animales de compañía, ya que podría ser un potencial problema veterinario. Se ha reportado el desarrollo de síndromes hemorrágicos en modelos con ratas y conejos ^5^^,^^16^, hallazgo que refuerza el enfoque de *One Health*.

En conclusión, este reporte de un caso de envenenamiento por orugas de *Lonomia* spp. en la Amazonía ecuatoriana alerta a médicos generales, dermatólogos y epidemiólogos de la región sobre la necesidad de considerar esta afección como un diagnóstico diferencial y conocer su manejo adecuado. Además, motiva a investigar sobre la distribución biogeográfica de las especies de orugas y polillas presentes en el país, y a indagar sobre los procesos de obtención y distribución del suero antilonómico.
